# Snoring induced reflux (SnoR) and a carcinoma in the gastro-oesophageal junction

**DOI:** 10.1515/iss-2021-0011

**Published:** 2022-06-01

**Authors:** Gunnar Loske

**Affiliations:** Department for General, Abdominal, Thoracic and Vascular Surgery, Katholisches Marienkrankenhaus Hamburg gGmbH, Hamburg, Germany

**Keywords:** AEG, cancer, endoscopy, negative pressure, oesophagus, reflux

## Abstract

Little is known about the relationship between snoring and the development of carcinomas of the gastro-oesophageal junction. The aim of the paper is to draw attention to snoring induced reflux (SnoR) observed during a routine preoperative examination in a patient with AEG type II after neoadjuvant chemotherapy. During the examination the patient (body mass index 26.5) started to snore deeply. As a consequence, remarkable morphological-functional changes at the gastro-oesophageal junction became apparent. Reflux was periodically aspirated, accompanied by snoring on inspiration. In our patient, the carcinoma developed precisely at this point of contact. The phenomenon of SnoR is demonstrated in a video. SnoR could be an explanation for the described correlation between snoring and the occurrence of carcinomas in the gastro-oesophageal junction. Further studies on the phenomenon of SnorR could be of major interest for the understanding of AEG carcinomas. It is possible that new preventive measures can be derived from these findings.

Little is known about the relationship between snoring and the development of carcinomas of the gastro-oesophageal junction [1, 2]. The aim is to draw attention to snoring induced reflux (SnoR) observed during a routine preoperative endoscopical examination.

After completion of neoadjuvant chemotherapy, a 64-year-old patient was referred for esophagectomy. Initially, a T3N2 AEG type II adenocarcinoma had been diagnosed. No evidence of a Barrett esophagus was preexisting. Preoperative restaging had shown impressive local remission without distant metastasis. Resection was recommended after the decision of the interdisciplinary tumor board. In preparation for surgery, a preoperative esophagogastroscopy was performed in the left lateral position with CO2 insufflation under sedation (Figure 1).

**Figure 1:**
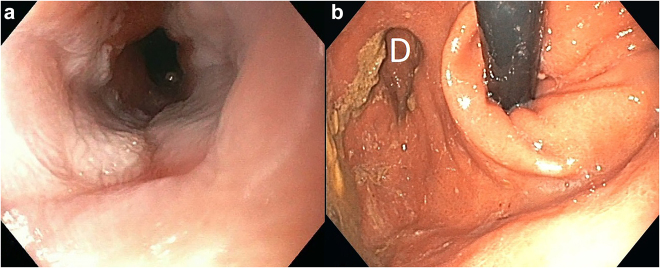
Anatomy of the gastro-esophageal junction without snoring. a: orthograde, b: Retrograde view. Incidental finding: Diverticulum (D).

During the examination the patient (body mass index 26.5) started to snore deeply. As a consequence, remarkable morphological–functional changes at the gastro-esophageal junction became apparent. With each inspiration movement, the distal sphincter relaxed, the hiatus dilated and gastric mucosa with reflux was sucked into the distal oesophagus (Figure 2). Reflux was periodically aspirated, accompanied by snoring on inspiration (Figure 3). In the orthograde view, the prolapse and SnoR was more pronounced after decompression of the stomach.

**Figure 2:**
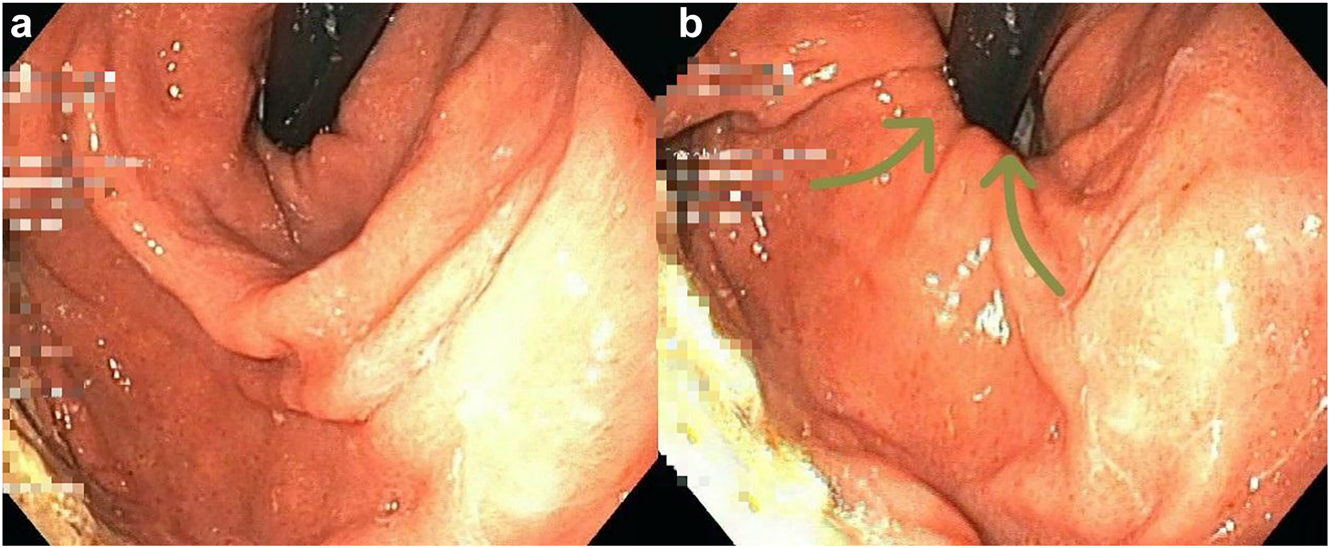
From a to b: The gastro-esophageal junction with snoring during breathing in from retrograde view. b: Hiatus opens and the gastric mucosa is sucked in (arrows).

**Figure 3:**
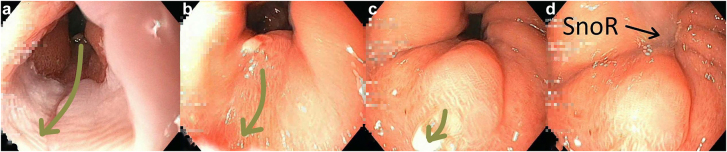
From a to d: The movement of the gastric mucosa (arrows) during the snoring from orthograde view. d: Snoring induced reflux (SnoR) is aspirated in the esophageal lumen.

Snoring-induced airway obstruction during inspiration causes increased intrathoracic negative pressure. This means that the already physiologically present inspiratory negative pressure is once again lowered significantly during the inspiration movement. As a result, gastric mucosa and reflux are actively aspirated into the distal esophageal lumen (Figures 2 and 3). Relaxation of the distal sphincter and dilatation of the hiatus opening will promote this effect. In our patient, the carcinoma had developed precisely at this point of contact. The phenomenon of SnoR is demonstrated in Video 1.

**Figure:**
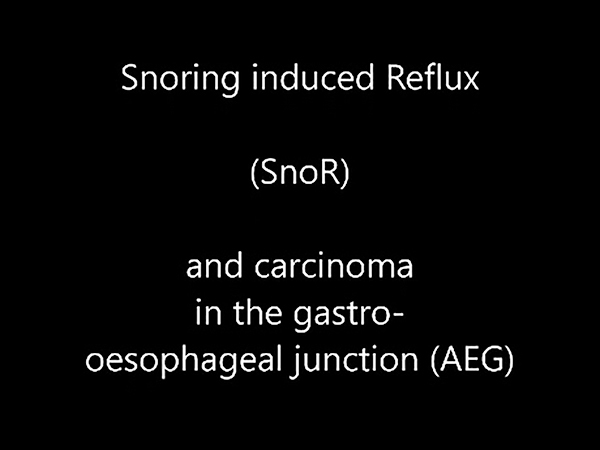


**Video 1:** The video demonstrates the phenomenon of the Snoring induced Reflux (SnorR): On endoscopic examination, the patient began to snore deeply. Morphological-functional changes became apparent. Gastric mucosa and reflux were sucked in the oesophagus during inspiration. The carcinoma had developed precisely at this point of contact.

SnoR could be an explanation for the described correlation between snoring and the occurrence of carcinomas in the gastro-esophageal junction. Further studies on the phenomenon could be of interest. SnoR should be examined with pH impedance and endoscopy in a sleep laboratory. It may be possible to derive targeted therapeutic measures from further evaluation of the observation described, such as a recommendation for nocturnal acid blockade or other preventive methods.

## Supplementary Material

Supplementary Material
