# Supplementary material for: Snoring induced reflux (SnoR) and a carcinoma in the gastro-oesophageal junction
Source: Innov Surg Sci. 2022 Jun 1;7(1):1–3. doi: 10.1515/iss-2021-0011 (PMC9352186; doi:10.1515/iss-2021-0011)
Supplement: Supplementary file 3 — Supplementary Material [file j_iss-2021-0011_suppl.pdf]

Reviewer Assessment

Loske G.: Snoring induced reflux (SnoR) and a carcinoma in the gastro-oesophageal junction

Reviewers' Comments to Original Submission

Reviewer 1: anonymous

Date received: 06-May-2021
Reviewer recommendation: **Return to author for minor modifications**
Reviewer overall scoring: **High**

Assessment Form scores: 5 = High/Yes; 3 = Medium/Adequate; 1 = Low

Is the subject area appropriate for the journal		4			
Does the title clearly reflect the paper's content?	5				
Does the abstract clearly reflect the paper's content	5				
Do the keywords clearly reflect the paper's content?	5				
Does the introduction present the problem clearly?	5				
Are the results/ conclusions justified?			3		
How comprehensive and up-to-date is the subject matter presented?		4			
How adequate is the data presentation?					
Are units and terminology used correctly?				2	
Is the number of cases adequate?					1
Are the experimental methods/ clinical studies adequate?					
Is the length appropriate in relation to the content?	5				
Does the reader get new insights from the article?	5				
Please rate the practical significance.			3		
Please rate the accuracy of methods.				2	
Please rate the statistical evaluation and quality control.					
Please rate the appropriateness of the figures and tables.	5				
Please rate the appropriateness of the references.		4			
Please evaluate the writing style and use of language.		4			
Please judge the overall scientific quality of the manuscript.			3		
Are the methods used worthy of reproduction in greater deal?	Yes				
Would you be willing to review a revision of this manuscript?	yes				

Comments to author: Interesting report. Please add an expressiv declaration that no evidence of a Barretts oesophagus is pre-existing (in the video it doesnt look so).

Reviewer 2: Meier, Peter

Date received: 26-May-2021
Reviewer recommendation: **Reject for the following reasons**
Reviewer overall scoring: **Unacceptable**

Assessment Form scores: 5 = High/Yes; 3 = Medium/Adequate; 1 = Low

Is the subject area appropriate for the journal					1
Does the title clearly reflect the paper's content?			3		
Does the abstract clearly reflect the paper's content					1
Do the keywords clearly reflect the paper's content?			3		
Does the introduction present the problem clearly?		4			
Are the results/ conclusions justified?					1
How comprehensive and up-to-date is the subject matter presented?				2	
How adequate is the data presentation?					1
Are units and terminology used correctly?		4			
Is the number of cases adequate?					1
Are the experimental methods/ clinical studies adequate?		4			
Is the length appropriate in relation to the content?		4			
Does the reader get new insights from the article?				2	
Please rate the practical significance.				2	
Please rate the accuracy of methods.					1
Please rate the statistical evaluation and quality control.					1
Please rate the appropriateness of the figures and tables.		4			
Please rate the appropriateness of the references.				2	
Please evaluate the writing style and use of language.			3		
Please judge the overall scientific quality of the manuscript.					1
Are the methods used worthy of reproduction in greater deal?					
Would you be willing to review a revision of this manuscript?	no				

Comments to author: The authors describe a typical phenomenon of pronounced inhalation and exhalation as it can occur physiologically in principle but pointedly in snoring. It is problematic to establish a connection between this casuistic observation and carcinomas of the gastroesophageal junction, only a limited literature data base is available.The authors correctly conclude that for such a correlation, e.g., a pH-metry would be necessary, but they have not listed it. Also the basic recommendation of a nocturnal acid blockade has to be evaluated critically. Certainly, the title "Snoring induced reflux and carcinoma in the gastro-oesophageal junction" can at best be considered as an indication of a fall.

Authors' Response to Reviewer Comments

Date received: 28-Jan-2022

Response to reviewer 1

I thank you for the interest. There was no evidence of Barrett's esophagus. This was inserted in the manuscript text:

No evidence of a Barrett oesophagus was preexisting.

Response to reviewer 2

I agree with the statement and thank for the important advice.

I did not want the article to be understood in such a way that already statements for a general therapeutic medicamentous recommendation should be derived from it. It is an observation of a single case. It is more about drawing attention to this phenomenon in snoring, which I had not noticed before in this clear expression and in connection with an AEG carcinoma.

I've adjusted the last few sentences of the text a bit, so hopefully it's more moderate and clearer now.

Manuscript was changed with:

SnoR should be examined with pH impedance and endoscopy in a sleep laboratory. It may be possible to derive targeted therapeutic measures from further evaluation of the observation described, such as a recommendation for nocturnal acid blockade or other preventive methods.

Comments by the Editor-in-Chief to Revised Submission

Dear reader,
Despite two different review reports and in light that we asked several other experts for an additional review, but without any response, we decided to publish the article and the video. First of all, the author responded to the criticism of the reviewers and second, we feel that this is indeed an interesting observation with respect to the development of carciomas at the GE-junction. Since these tumors show a constant rise and morbid obesity is often associated with snoring, we hope that this report may result in further studies to evaluate a possible correlation. Letters to the editor are highly welcome and appreciated.

Best regards, Joachim Jaehne on behalf of the Editorial Board
